# Complete Chloroplast Genome Sequence of a White Spruce (Picea glauca, Genotype WS77111) from Eastern Canada

**DOI:** 10.1128/MRA.00381-19

**Published:** 2019-06-06

**Authors:** Diana Lin, Lauren Coombe, Shaun D. Jackman, Kristina K. Gagalova, René L. Warren, S. Austin Hammond, Heather Kirk, Pawan Pandoh, Yongjun Zhao, Richard A. Moore, Andrew J. Mungall, Carol Ritland, Barry Jaquish, Nathalie Isabel, Jean Bousquet, Steven J. M. Jones, Joerg Bohlmann, Inanc Birol

**Affiliations:** a Canada’s Michael Smith Genome Sciences Centre, BC Cancer, Vancouver, BC, Canada; b Department of Forest and Conservation Sciences, University of British Columbia, Vancouver, BC, Canada; c British Columbia Ministry of Forests, Lands and Natural Resource Operations, Tree Improvement Branch, Kalamalka Forestry Centre, Vernon, BC, Canada; d Laurentian Forestry Centre, Natural Resources Canada, Quebec City, QC, Canada; e Canada Research Chair in Forest Genomics, Université Laval, Quebec City, QC, Canada; f Michael Smith Laboratories, University of British Columbia, Vancouver, BC, Canada; Vanderbilt University

## Abstract

Here, we present the complete chloroplast genome sequence of white spruce (Picea glauca, genotype WS77111), a coniferous tree widespread in the boreal forests of North America. This sequence contributes to genomic and phylogenetic analyses of the *Picea* genus that are part of ongoing research to understand their adaptation to environmental stress.

## ANNOUNCEMENT

Over tens of millions of years, conifers such as the white spruce (Picea glauca) have evolved to cope with adverse environmental conditions (1, 2), such as prolonged drought and increased pressure from forest insect pests (3). Plants have three different genomes, namely, a nuclear, a mitochondrial, and a plastid (i.e., chloroplast) genome. In general, chloroplast genomes are derived from the ancestral genomes of the microbial endosymbiont from which these organelles originated (4). The nuclear genome of *P. glauca* (genotype WS77111) was published in 2015 (5).

A *P. glauca* (genotype WS77111) needle tissue sample was collected in southeastern Ontario (44°19′48″N, 78°9′0″W; elevation, 250 m). Genomic DNA was extracted from 60 g of tissue by Bio S&T using an organelle exclusion method yielding 300 μg of high-quality purified nuclear DNA, as previously described (6). The sample was sequenced at Canada’s Michael Smith Genome Sciences Centre (GSC). Here, we report on the assembled and annotated chloroplast genome sequence of this genotype.

To sequence the sample, genomic DNA libraries were constructed according to the plate-based and paired-end library protocols at the GSC on a Microlab Nimbus liquid-handling robot (Hamilton, USA). Briefly, 1 μg of genomic DNA was sonicated (Covaris LE220) in 62.5 μl to 400 bp and purified with PCRClean DX magnetic beads (Aline Biosciences). Illumina sequencing adapters were ligated overnight at 16°C. Pooled libraries were sequenced with paired-end 250-bp reads on an Illumina HiSeq 2500 instrument in rapid mode. Using this protocol, four libraries were generated, sequencing approximately 400 million reads from each one.

To assemble this genome sequence, we generated various random subsamples of read pairs from one lane of one library (i.e., 42,881,319 read pairs), producing subsets with 21×, 43×, 88×, 172×, 345×, 711×, 1,219×, and 5,619× coverage of the chloroplast genome. Each subset was assembled with ABySS v2.1.0 (7) (using the parameters *k* = 128 and *kc* = 3). Due to the large number of chloroplasts per cell, the chloroplast genome would be sequenced at a very high coverage over a full lane of data. Therefore, by subsampling the full data set, the coverages of the nuclear and mitochondrial genomes were lowered to a level where these sequences do not assemble well, while the coverage of the chloroplast genome was still sufficient enough for a high-quality assembly. The 43×, 88×, and 172× subsets produced the best ABySS assemblies (*N*_50_ lengths, 3,692, 1,313, and 949 bp, respectively), as determined by a QUAST analysis (v5.0.0) (8). For comparison, we used the white spruce admix (PG29) chloroplast genome (NCBI GenBank accession number NC_028594) (9), the published chloroplast genome that is most closely related to the WS77111 genotype. The use of this admix as a reference was established previously (10), as it is a naturally occurring ingress of *Picea glauca*, Picea engelmannii, and Picea sitchensis (5). We then performed additional ABySS assemblies with various *k* and *kc* parameters using these three subsets (*k* = 96, 112, 128, 144, and 160; *kc* = 3 and 4). The assembly with the fewest aligning contigs (*n* = 14) and fewest misassemblies (43×; *k* = 96; *kc* = 3) was chosen for further scaffolding with the PG29 chloroplast genome, using LINKS v1.8.5 (11), thereby joining the contigs into one piece. We then used Sealer v2.1.0 (12) to close the scaffold gaps. To be consistent with previously published chloroplast genomes when reporting gene annotations, we adjusted the start position of our assembly using BLAST v.2.7.1 (13) and polished the final assembly with Pilon v1.22 (14), using BWA v0.1.7 (15) for read alignment.

**FIG 1:**
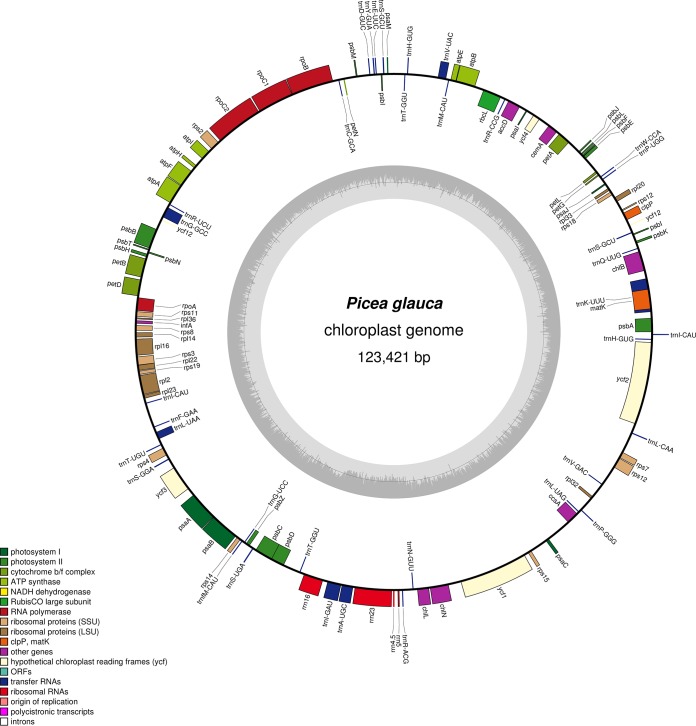
Complete chloroplast genome of *Picea glauca* genotype WS77111. The *Picea glauca* chloroplast genome was annotated using GeSeq v1.65 (16) and plotted using OrganellarGenomeDRAW v1.2 (17). The inner gray circle illustrates the G+C content of the genome.

The complete genotype WS77111 chloroplast genome is 123,421 bp long, with a G+C content of 38.74%. Using GeSeq v1.65 (16) with several *Picea* sp. chloroplast genomes as references (9, 10), we annotated 114 genes, namely, 74 protein-coding, 36 tRNA-coding, and 4 rRNA-coding genes. Five genes (*rps12*, *petB*, *petD*, *rpl16*, and *psbZ*) required manual annotation. The genome map in Fig. 1 was generated using OrganellarGenomeDRAW v1.2 (17).

The assembly of this new chloroplast genome will enable further analysis of the phylogeny and genetics of *Picea* spp.

### Data availability.

The complete chloroplast genome sequence of *Picea glauca*, genotype WS77111, is available in GenBank under accession number MK174379, and the raw reads are in the SRA under accession numbers SRX525336 and SRR1259605. The annotations used as references were from Picea abies (GenBank accession number NC_021456), Picea asperata (GenBank accession number NC_032367), *Picea glauca* genotype PG29 (GenBank accession number NC_028594), Picea morrisonicola (GenBank accession number NC_016069), and Picea sitchensis (GenBank accession numbers NC_011152 and KU215903).
